# Students’ perception of online learning amidst the Covid-19 pandemic: A study of junior, senior high school and college students in a remote area

**DOI:** 10.12688/f1000research.52152.2

**Published:** 2022-02-14

**Authors:** Senida Harefa, Grace Lamudur Arta Sihombing

**Affiliations:** 1 Faculty of Religious Education, Christian Education Management, Institut Agama Kristen Negeri Tarutung, Tarutung, Sumatera Utara, 22758, Indonesia

**Keywords:** students' perception, online learning, COVID-19 pandemic

## Abstract

**Background: **The COVID-19 pandemic has brought about many changes in all sectors of life, especially in the field of education. These changes aim to make the learning process more effective in the pandemic environment. However, it can be challenging, as some students do not give positive responses to these changes, especially those in remote areas. This article aims to identify and report students' perceptions about the effectiveness of online learning during the COVID-19 pandemic in the remote North Tapanuli region of Indonesia.

**Methods: **In this study, data were obtained using an online survey involving 30 students from three levels of education, namely junior high school, senior high school, and college. They are students who the teacher appoints as class administrators; through them, the teacher communicates about everything—learning activities. So compared to his other friends, they know more about the implementation of online learning during the covid 19 pandemic. In addition, schools in remote areas have fewer students than schools in big cities. So we conclude that this research is considered representative with thirty students as the sample. The data gathered from the survey were analyzed using quantitative descriptive methods.

**Results:** Results show that online learning is considered less effective by students in remote areas; this happens because communication networks and infrastructure do not adequately support them to follow online learning.

**Conclusion: **Teachers need to evaluate how to teach as well as re-design models and approaches to be applied in learning. This can be achieved by adjusting to the student’s current situation to generate interest and willingness to learn online.

## Introduction

The COVID-19 pandemic has had a major impact on various aspects of peoples’ lives, namely in the economic, socio-cultural, and educational aspects. It is a global problem affecting educational institutions. Since the start of this pandemic, it has caused shock and disruption to students. The pandemic has forced schools to close and lessons that were carried out face-to-face have shifted to the online world. The use of the Internet and many other significant technologies to create materials for educational purposes, educational distribution, and program management constitute online learning ( Fry, 2001). All educators are asked to make a transition, due to the closure of school buildings. There is no other choice but to apply online learning; even though many feel unprepared during this transitional period, students must adjust themselves while trying to build meaning amid various challenges related to the pandemic. Even though learning is carried out online, it is hoped that learning outcomes will remain maximal. There is some evidence that online learning can lead to higher student success ( Kurucay & Inan, 2017). A great amount of evidence indicates that there is no substantial difference in the efficacy of well-designed online learning relative to well-designed face-to-face learning ( Clark, 2007).

However, the reality is not as expected since not all students respond positively to the implementation of online learning. Today, the majority of colleges and universities still face virtual learning difficulties ( Talidong & Toquero, 2020). For example, not all educators and students can use e-learning applications, especially those in remote areas. They feel that they are not optimal in learning. During online learning, they deal with several obstacles such as more assignments that make them feel burdened. This happens since teachers or lecturers in charge assign them two or three tasks for every lesson. Additionally, network connection disturbance in rural areas affects their attendance of online learning. Online learning also influences the students’ motivation in doing assignments. Therefore, the objectives of online learning goals are not always achieved effectively. Students who succeed in learning are those who are active and always follow the learning. Parents of students also confirmed that their children were too lazy to learn online. Most of them show unsatisfactory results. For example, performance in learning is decreased compared to face-to-face learning, Some students quit school and do not continue their education. This situation gives a bad picture of the learning attitudes of students.

In Anna Ya Ni’s it is suggested that the use of the video chat software Zoom has the greatest potential to improve classes in order to meet student concerns. Zoom is one of the most frequently used applications in online learning to replace conventional face-to-face classes ( Ni, Wart, Medina, Collins, Kimberly, & Pei, 2020). It turns out that technology is used as a tool or media in learning which cannot replace face-to-face interactions between teachers and students ( Miller, 2020). The obstacles of online learning in rural areas encourage researchers to conduct this research. Therefore, the objective of this research is to identify the students’ perception of the effectiveness of online learning. The researchers wanted to gain a deep and detailed understanding of students' online learning experiences, Based on the support they gained during the learning process and satisfaction with the learning facilities used. The findings of our research, both strengths and weaknesses, will be valuable inputs for institutions to improve the quality of distance learning by adjusting to the needs and conditions of students in remote areas.

## Literature

The development of information and communication technology at this time provides many benefits for human life, so the mastery of such technology is no longer an option but has become a necessity. Through the existence of Internet networks, the use of technology in the educational environment has opened new avenues for educators; face-to-face learning has been transformed into e-learning or online learning ( Bernard *et al*., 2009). In addition to other electronic media, such as CD-ROM, satellite, and television, some experts classify e-learning as ‘education delivered via the Internet’, while online education is described as ‘education delivered only via the Internet or web-based media’ ( Lee, 2017). When used interchangeably, online education or e-learning is commonly defined as bridging the space between teachers and students through the use of web-based technology ( Ryan & Young, 2015).

The presence of the Internet facilitates human work in many ways, especially in the field of education. The current learning process requires teachers and students to use technology. However, not all students can accept and adapt to these changes. The acceptance of changes in the learning process differs among students. This can be influenced by age, thinking ability, and students’ interest in technology. Students of all ages seem to react differently to the practice of online learning, with older students showing greater appreciation. There are still major variations in how learners view their online interactions during learning ( Koohang, Paliszkiewicz, Nord, & Ramim, 2014). There are also concerns about the online learning environment’s efficacy ( Hashem, 2011).

Students’motivation in taking online learning can be assessed by how they participate in ongoing learning. Participation in online learning requires three dimensions, namely cognitive participation, emotional participation, and behavioral participation ( Fredricks, Blumenfeld, & Paris, 2004). These three dimensions are explained as follows: (1) Cognitive participation is the cognitive effort of a student to acquire skills in the online learning process. (2) Emotional participation is described as students’ positive emotions towards teachers, peers, and online learning. (3) Behavioral participation is participation that is manifested by activities that pay attention to learning when studying online ( Jung & Jeongmin, 2018).

Motivation is generated through effective learning. Effective online learning requires effective instructional design and procedures based on the concept of a good education. It will positively impact the instructions if the plan is correct. For example, designing learning must consider the method, flexibility of time, student involvement in activities, and presentation of material ( Simonson *et al*., 2015; Barberà *et al*., 2016). Variations in teachers' teaching style and their expertise in using technology in interacting with students also significantly affect the Motivation and results of e-learning ( Ozkan & Koseler, 2009). Several studies on using technology can change the learning experience and improve student performance, such as posting videos after class and using social media platforms such as Facebook, YouTube, and other e-learning tools. The effect on perceived satisfaction and effectiveness online is when teachers have sufficient and up-to-date knowledge in their area of expertise ( Early & Murphy, 2009). The increasing use of technology in online learning benefits students, but the main concern is unstable internet connections in remote areas, not providing comfort in learning ( Kulal & Nayak, 2020). Based on the description above, we developed a research instrument from the aspects: Teachers’ methods of online learning, Students’ convenience in online education, Motivation to learn online, and The effectiveness of online learning, to gain a detailed understanding of students’ experiences of online learning during COVID 19.

## Methods

An online-based questionnaire study was conducted in a remote area, North Tapanuli, Indonesia. The main objectives of this study were as follows:

1. To assess students’ perception of the effectiveness of online learning during the COVID-19 pandemic using four indicators: 1) Teachers’ methods of online learning. 2) Students’ convenience in learning online. 3) Motivation to learn online. 4) The effectiveness of online learning.
2. To find out the differences in average perception scores about online learning between three groups of students: 1) Junior high school students. 2) Senior high school students. 3) Students from college in a remote area.

### Ethics

This research project was approved by the Research Ethics Committee. Ethical Approval Involving Human Respondent from tertiary education (Approval number: 1437.1/Ikn.01/TL.01/09/2020), from junior high school education (Approval number: 086/SMP-SM/IX/2020), and senior high school education, (Approval number: 422.1/063/SMA N 1TRT/2020). Written informed consent from all subjects involved was obtained for participation in the study and subsequent publication.

### Data collection

Primary data was collected through an online survey (see Table 1). The survey included 20 items on a four-point Likert scale, from 1 (disagree), 2 (neutral), 3 (agree), 4 (strongly agree). The survey was conducted for over a week. Students were asked to participate in a web-based survey. Of the 75 students surveyed, only 30 students submitted their answers to the online survey, namely 10 students from junior high school education, 10 students from senior high school education and 10 undergraduate students from tertiary education. They are students who are appointed by the teacher as class administrators, which acts as a regulator of the class, connecting students and teachers field of study, and through them every teacher to communicate to all the learning activities, such as setting the learning time, record the attendance of students in the classroom, and also reports on student assignments in one class, submitted to the subject teacher through them. They are the ones who know a lot about the implementation of learning during the Covid 19 pandemic, compared to other friends. Schools and students in remote areas are few, not as many as schools and students in big cities, so we conclude that the thirty samples are considered representative of the sample in this study. In this case, gender demography is an important factor to be analyzed.

**Table 1.** Frequency of respondent statistics.

No	Item	1	2	3	4	N	Score	Mean	%	Category
Teacher’s methods in online learning										
1	Obtain and find out the teaching materials/learning materials delivered by the teacher/lecturer when studying online. Good	0	0	11	19	30	109	3.63	90.83%	Effective
2	Understanding of the material presented by the teacher/lecturer when studying online studied	1	5	18	6	30	89	2.97	74.17%	Less effective
3	Can re-describe the material that has been online by the teacher/lecturer on time	2	4	17	7	30	89	2.97	74.17%	Less effective
4	Responding to questions that appear in discussion forums of subject matter provided by the teacher/lecturer during online learning	3	5	21	1	30	80	2.67	66.67%	Less effective
5	Apply the subject matter delivered by the teacher/lecturer in everyday life	4	5	16	5	30	82	2.73	68.33%	Less effective
Students’ convenience in online learning										
6	Can communicate smoothly with the teacher/lecturer during online learning	1	2	15	12	30	98	3.27	81.67%	Less effective
7	Can ask directly to the teacher/lecturer when I don’t understand the subject matter during online learning	0	3	18	9	30	96	3.20	80.00%	Less effective
8	Always get a good response from the teacher/lecturer during online learning	4	3	16	7	30	86	2.87	71.67%	Less effective
9	Enjoy doing assignments given by the teacher/lecturer on online learning	4	7	16	3	30	78	2.60	65.00%	Less effective
10	Feel comfortable because the teacher/lecturer always understands the obstacles experienced when learning online (for example network barriers and data packets)	1	9	15	5	30	84	2.80	70.00%	Less effective
11	Active in following class discussion forums created by the teacher/lecturer during online learning	1	8	16	5	30	85	2.83	70.83%	Less effective
Learning Motivation in online learning										
12	Always on camera during online learning	2	7	17	4	30	83	2.77	69.17%	Less effective
13	Pay attention when teacher/lecturer provides learning explanations during online learning	1	4	22	3	30	87	2.90	72.50%	Less effective
14	Participate in discussion group study assignments formed by the teacher/lecturer	2	6	17	5	30	85	2.83	70.83%	Less effective
15	Submit assignments given by the teacher/lecturer on time	1	3	16	10	30	95	3.17	79.17%	Less effective
16	Learn guidelines about learning online from the internet	1	12	15	3	30	81	2.70	67.50%	Less effective
17	Sit calmly during online learning in front of the laptop/cellphone until the time set by the school/teacher/lecturer elapses	0	5	22	3	30	88	2.93	73.33%	Less effective
Effective online learning										
18	Likes online learning rather than face-to-face learning	0	9	16	5	30	86	2.87	71.67%	Less effective
19	The interaction of online teaching and learning is better than face-to-face learning	3	9	15	3	30	78	2.60	65.00%	Less effective
20	Online learning facilities always support, both in terms of equipment (for example mobile/laptop) or network.	0	7	14	9	30	92	3.07	76.67%	Less effective
Mean									72.96%	Less effective

### Instrument

Data in this study were collected through the use of questionnaires. Questionnaires consisted of four indicators; 1) Teachers’ methods of online learning; 2) Students’ convenience in online learning; 3) Motivation to learn online; 4) The effectiveness of online learning. Then the indicators were translated into 20 questionnaire items ( Table 1).

### Statistical analysis

Data were collected, coded, checked for completeness and input into SPSS Version 25 IBM (SPSS Statistics, RRID:SCR_019096). R is an open-source alternative software that can also be used to do the same analysis. Descriptive statistics (frequency, percentage, mean and standard deviation) were used to describe variables. One-way analysis of variance (ANOVA) was used to determine differences in perceptual scores about learning online for junior high school, senior high school, and college students. In all experiments in this report, we applied an alpha level of. 05.

## Results

20 questionnaires, which were elaborated on 4 indicators, were used to survey students' experiences in online learning. Based on the results of the calculation of the data obtained, the value of each questionnaire indicator was as follows: The teacher’s method in online learning (score = 89.8; average = 2,992; percentage = 74.83%), student comfort in online learning (score = 87.83; average = 2,928; percentage = 73.19%), learning motivation in online learning (score = 86.5; average = 2.883; percentage = 72.08%), effective online learning (score = 85.33; average = 2.846; percentage =71, 11%). After being calculated, the average percentage score = 72.96%. So, based on the hypothesis H1: p ≥ 85% (effective), H0: p ≤ 85% (less effective) indicates that students’ perceptions towards online learning in remote areas are less effective. The shortcomings are related to Teachers' methods of online learning; Students' convenience in online learning; Motivation to learn online; The effectiveness of online learning. The four indicators must be improved so that students' motivation, performance, and achievement in education can increase optimally ( Table 1).

Using the One Way ANOVA test, the researchers aim to figure out the difference in the percentage of perceptions between the three groups of students. The conditions that must be met to process data in a One-way ANOVA test are the data must be normally distributed, and the variance must be homogeneous. After our data were processed, the normality test met the first of these requirements, namely, a significance value of.103 > 0.05 (Shapiro-Wilk) thus the data was declared to be normally distributed.

The second step is to test the homogeneity. Results of the homogeneity of variance test obtained a significance value of.093 > 0.05. Thus, we can be confident that our data distribution was homogeneous.

The output in the descriptive section shows the average value of students’ perceptions about online learning: student at junior high school (mean) = 58.10, student at senior high school (mean) = 55.30 and college student (mean) = 61.70. The highest score stating that online learning is less effective than face-to-face learning is that of college students, n = 30, 95% confidence interval for mean, total min = 48 and max = 73.

The mean scores of each group are different in number, even though the difference is not significant. Meaning that students from the three groups have learning experiences that are not much different in online learning, junior high school, senior high school and college students.

The ANOVA output in the descriptive section shows sum of squares total = 724,967; df = 29; mean square = 102,933 and 19.226; F = 5.354 and a significance value of 0.011 <0.05, meaning that the average value of student perceptions of the three levels of education about online learning is not significantly different. Students as a whole report the same perception that online learning is less effective in a remote area ( Table 2).

**Table 2.** Descriptive statistics for variables.

Descriptives								
Perception								
	N	Mean	Std. deviation	Std. error	95% confidence interval for mean		Min	Max
					Lower bound	Upper bound		
Junior high school	10	58.10	3,814	1206	55.37	60.83	48	61
Senior high school	10	55.30	2,710	.857	53.36	57.24	51	61
College	10	61.70	5,982	1892	57.42	65.98	54	73
Total	30	58.37	5,000	.913	56.50	60.23	48	73

Then, the authors conducted a follow-up ANOVA test using Duncan’s test to determine the perceived significance value between junior high school with senior high school students and senior high school with college students. Duncan’s test results have two subsets, namely in the first subset, the significance value was 0.077 > 0.005 of senior and junior high school students’ perceptions, meaning that their perceptions about online learning are not significantly different. In the second subset, the significance value was 0.165 > 0.05 of senior high school and college students’ perceptions, meaning that their perceptions about online learning were also not significantly different. So, the results of Duncan’s test concluded that there was no significant difference between students’ perceptions of online learning, meaning that they had the same perception ( Table 3).

**Table 3.** Advanced test of ANOVA (perception of Duncan about student perception).

Perception of Duncan			
Education level	N	Subset for alpha = 0.05	
		1	2
Junior high school	10	55.30	
Senior high school	10	58.10	58.10
College	10		61.70
Significant		.077	.165

The means of groups in homogeneous subsets are displayed. Uses harmonic mean sample size = 10,000.

In this study, more male students answered that online learning was less effective than female students. The result of data calculation showed that the frequency of male students’ answers was 66.7%, while the frequency of female students’ answers was 33.3%.

## Discussion

The overall mean score obtained in this study p = 72,96% thus (H0:72, 96% ≤ 85%) indicates that students’ perception of online learning in remote areas is that it is less effective than face-to-face learning. The resulting score needs to be improved for the achievement of learning objectives. To enable teaching activities to be carried out at an adequate level, the constraints to be considered are:

1. Teacher teaching methods in online learning
  This study has problems related to teacher teaching methods in online learning. Students feel that the technique used by the teacher is very monotonous. In general, teachers only use the assignment method. The assignment method is a one-way learning pattern; students require no teaching and learning interaction. Tedious learning methods are considered less effective because they make learning passive. The use of effective learning methods or strategies can improve student academic achievement ( Donker *et al*., 2013). Implementing learning, teachers must consider the use of teaching methods. The methods used should vary. Variations in the learning process are variations in the presentation of learning activities. Nowadays, technology offers a variety of learning methods that facilitate students to learn and do the assignments conveniently ( Pasaribu *et al.*, 2020). The application of various teaching methods can create creativity in learning and can eliminate boredom in students. There are five items regarding teaching methods, namely knowing, understanding, responding, describing and applying. The five items are inputs for the teachers so that they can design and review the online learning that has been implemented so far. The purpose of learning is to instill knowledge in students; whether good or not, students’ acceptance of the material presented depends on the method used by the teacher in learning. This also cannot be separated from the teacher’s own knowledge. The more knowledgeable a teacher is, the better he or she will be in conveying learning to their students. With the implementation of varied learning activities, it is hoped that learning will be more meaningful and optimal. Students consistently show perseverance, enthusiasm and fully participate in learning activities ( Rusman, 2017).
2. Comfort of students in online learning
  The results of this study stated that students are less comfortable with online learning. The feeling of inconvenience represents dissatisfaction. For example, communicating with teachers is often hampered by unstable networks, and abilities in using the technology are still limited resulting in delays in joining lessons. Another regrettable thing is that most teachers do not understand the barriers that prevent the start of online learning or that affect it while it is ongoing. This may affect the effectiveness of online learning. In response to this, it is necessary to implement blended learning in the future, which combines online learning with traditional physical classroom teaching. It aims to enlarge the learning method in education areas. During the pandemic, the implementation of blended learning might occur in certain remote areas in Indonesia. The pandemic situation could be controlled because of the less density of population in remote areas and also by the strict application of health protocols: washing hands frequently, wearing masks all the time, and keeping a distance from one another ( Garrison & Kanuka, 2004). Many other academic reports have confirmed the beneficial impact of blended learning and can increase academic success ( Alipour, 2020). In addition, the use of blended learning can increase student engagement to stay active in learning, thereby strengthening their role in their education as learners ( Mosca, 2010; Vernadakis *et al.*, 2011). So the problem of inconvenience in online learning can be overcome by implementing blended learning so that even amid COVID 19, education is still effective.
3. Learning Motivation in online learning
  Motivation is the most important factor in learning. Motivation affects the achievement of student learning success and serves as an impetus to carry out learning activities. There are two types of learning motivation. The first is extrinsic motivation, which refers to all factors from outside that play a role in achieving learning goals such as facilities, teachers, and the process of implementing the learning. And intrinsic motivation is a factor from the students themselves such as interest, feelings of pleasure, and desire ( Ryan & Deci, 2000). According to students in remote areas, online learning is less able to motivate students to learn. This is evidenced by students’ answers to the survey questions provided by the researchers. Students are not enthusiastic about online learning; they do not do assignments and do not submit assignments within the time that has been determined; they do not do study groups without the assistance of their teacher. This could be due to inadequate facilities, exhausted Internet packages or even students who cannot afford packages, and bad Internet network infrastructure. All these can cause a lack of motivation to learn in students. In summary, situations like this have a major impact on the way students learn and can lead to disappointing performance. Recent studies have shown that student engagement in virtual teaching is often of a lower quality than face-to-face teaching ( Dibner, 2020; Lan & Hew, 2020). This should get the teacher's attention. Teachers, as key holders in learning, can do various creative ways to make learning more interesting so that students are motivated to learn. In this case, it can be done, either through the use of various media, methods or learning models. All of them are important factors in making learning effective. This study contributes to the understanding that learning motivation is influenced by the extent to which learning is carried out effectively.
4. Effective online learning
  In this era, technology offers several advantages to assist human mobility practically. Besides, it also supports human communication and its efficiency, particularly the existence of cellular technology to facilitate rapid human connectivity ( Song, Karimi, & Kim, 2015). During the COVID-19 pandemic, all schools in Indonesia used the Internet network to send messages to students (online learning). In other words, online learning tools that include technology support the independent learning process ( Dunlap & Lowenthal, 2011). However, in using technology, it is also necessary to consider students’ perceptions. The results indicate that students in remote areas better recognized the effectiveness of face-to-face learning. As the aforementioned results related to the indicators suggest, students had several obstacles during online learning. The transformation of face-to-face into online learning affects the students’ learning process badly, for instance, the limitation of social interaction. In this case, the teacher is encouraged to think seriously about creative solutions to this problem to reach the teaching goals. E-learning plays an essential role in developing a meaningful educational experience for students. If well-designed materials and learning are implemented to the right technology and curriculum, it will produce satisfying learning goals ( Kidd, 2009). The positive impact of e-learning on student satisfaction lies in its functionality, reliability, ease of use, data quality, flexibility, portability, and integration ( Chiu, Chiu, and Chang 2007).
  According to the previous explanations, students generally have more fun when learning is done face to face. With face-to-face learning, students can directly get answers to their curiosity about the material being studied. After conducting this research, the assumptions about the displeasure or reduced effectiveness of online learning in this area were proved correct and significant. Times have changed. With the COVID-19 pandemic, students and teachers are required to use technology in learning since learning must now be done remotely to prevent crowds from gathering, to break the chain of the pandemic. Willingly or unwillingly, online learning must be practiced. The results of this research indicate that online learning is less effective according to the perception of students in remote areas.
  Experiences about online learning according to students' perceptions, were successfully collected through the use of the questionnaire method. Based on the survey results, it is known that there are some obstacles during online learning. The findings of this study lead us to consider the efforts made to improve motivation, performance, student involvement in learning, and expected achievement to be improved.
  The finding of this study can be used as a reference to improve the online teaching system. For teachers, it is helpful as self-evaluation to determine the level of success of the learning that has been carried out. Then figure out the students’ needs in lecturing, providing modules, applied methods, and learning media so that a vibrant learning atmosphere can be used and all students’ difficulties can be overcome. For parents, to provide an understanding regarding the importance of a comfortable learning environment for children. Cause there are still parents who have less knowledge regarding the difficulties their children experience in learning, especially those with low incomes because they are busy at work. Through this research, the parents recognize their children problems in education. Thus, parents support their children by assisting in learning and facilities, such as cellphones, laptops, internet packages, and other learning equipment. Furthermore, with the results of this study, students are expected to understand the current situation and online learning system, requires students to be independent in learning ( Ury & Ury, 2005; Geyer, 2007).
  From the frequency data, it is known that the response frequency was 66.7% by males and 33.3% by females, meaning that males responded more that online learning was not effective. Based on the results of data frequency, it is known that women’s motivation to learn online exceeds that of men. This is evident from the response of women to the tasks given by the teacher. In doing the tasks, women are much more disciplined than men. Also, women turn in assignments on time.

## Conclusions

The COVID-19 pandemic presents an extraordinary situation worldwide, this situation affects the implementation of learning in schools. Face-to-face teaching and learning interactions turn to the online world. The online learning method's challenges are practical activities that emphasize that effective learning requires designs and procedures based on appropriate education; teachers should not burden students with continuous assignments. Research shows that the failure of students to continue their education online in some cases due to the increased workload caused students to drop out of school ( Park & Choi, 2009). To achieve higher levels of academic success, teachers must ensure there is a complete curricular plan that is tailored to goals, avoiding a large number of student burdens that are practically impossible to meet ( Oliveira & Magalhães, 2020).

So, educators are expected to redesign implementing procedures for online learning so that students can still learn as much as possible. From the teacher's side, it is hoped that teachers will improve methods of teaching, by designing models and other approaches to provide variation in learning in order to raise students’ interest and willingness to learn online. Furthermore, each institution needs to prepare well for designing interesting learning media, and designing modules that are more flexible, making adjustments such that students adapt to changes in the teaching, learning and assessment, both face-to-face and online ( Ansari *et al.*, 2021), And blended learning is an effective teaching model amid pandemics to improve motivation, achievement, and learning performance. However, this is also a call for the government to improve internet networks and infrastructure in remote areas, in order to facilitate online education. The results of this research provide additional insight to all those involved in the implementation of education. However, further research is needed to obtain a more complete explanation.

## Data availability

### Underlying data

Figshare: Data survey about the effectiveness of online learning. https://doi.org/10.6084/m9.figshare.14191622.v1. ( Harefa & Sihombing, 2021).

This project contains the following underlying data.

• Research Data.xlsx (Questionnaire data in Microsoft Excel format).

Data are available under the terms of the Creative Commons Zero “No rights reserved” data waiver (CC0 1.0 Public domain dedication).
