# Supplementary material for: Students’ perception of online learning amidst the Covid-19 pandemic: A study of junior, senior high school and college students in a remote area
Source: F1000Res. 2022 Feb 14;10:867. Originally published 2021 Aug 27. [Version 2] doi: 10.12688/f1000research.52152.2 (PMC8844540; doi:10.12688/f1000research.52152.2)
Supplement: Peer review history

# Peer review: Reviewer response for version 2

**DOI:** 10.5256/f1000research.120634.r123458

**Published:** 2022-02-14

**Authors:** Xuesong (Andy) Gao

**Affiliations:** 1 School of Education, University of New South Wales, Sydney, NSW, Australia

I believe that the authors have addressed my reservations and I do not have any other issues with the study.

Is the work clearly and accurately presented and does it cite the current literature?

Partly

If applicable, is the statistical analysis and its interpretation appropriate?

Partly

Are all the source data underlying the results available to ensure full reproducibility?

Yes

Is the study design appropriate and is the work technically sound?

Partly

Are the conclusions drawn adequately supported by the results?

Partly

Are sufficient details of methods and analysis provided to allow replication by others?

Partly

Reviewer Expertise:

education

I confirm that I have read this submission and believe that I have an appropriate level of expertise to confirm that it is of an acceptable scientific standard.

# Peer review: Reviewer response for version 1

**DOI:** 10.5256/f1000research.55388.r93012

**Published:** 2021-12-08

**Authors:** Reza Rachmadtullah

**Affiliations:** 1 Department of Elementary School Teacher Education, Universitas PGRI Adi Buana, Surabaya, Indonesia

Overall, the authors have done a great job. It is a very important topic under the current circumstances. I do not think that the authors need to include the title of Anna Ya Ni’s research in this article. There is general relevant information regarding e-learning during Covid-19 and is very current. In this study, it is better to add the latest references related to the research objectives in the discussion section. In the conclusion of this study, the authors should add a paragraph about the implications and usefulness of the results in this study.

Is the work clearly and accurately presented and does it cite the current literature?

Yes

If applicable, is the statistical analysis and its interpretation appropriate?

Yes

Are all the source data underlying the results available to ensure full reproducibility?

Yes

Is the study design appropriate and is the work technically sound?

Yes

Are the conclusions drawn adequately supported by the results?

Yes

Are sufficient details of methods and analysis provided to allow replication by others?

Yes

Reviewer Expertise:

NA

I confirm that I have read this submission and believe that I have an appropriate level of expertise to confirm that it is of an acceptable scientific standard.

# Peer review: Reviewer response for version 1

**DOI:** 10.5256/f1000research.55388.r94627

**Published:** 2021-09-20

**Authors:** Xuesong (Andy) Gao

**Affiliations:** 1 School of Education, University of New South Wales, Sydney, NSW, Australia

In the abstract, I am unsure if the changes brought by the pandemic were aimed to make the learning process more effective. I think that the background section needs to explain why the study needed to examine the students’ perceptions. I am not sure if 30 students were sufficient to examine the perceptions of students related to online learning at the three levels. The results are not particularly surprising. Since the number of participants in the study is quite small, I was wondering if the authors can make robust claims about the findings and these findings have significant implications for practice.

**Introduction:** I suggest that the authors summarize what was found in studies on students during the pandemic. A large number of studies have been published on the topic but it is true that not much was done in relation to students in remote areas. I do not think that the authors need to include the details of Anna Ya Ni’s research (I mean, the article title).

**Literature review:** Why do the authors talk about students’ seriousness in online learning? Not their commitment, engagement, or motivation? The literature review really needs to elaborate on why we need a study on students’ perceptions. It should also motivate the research questions to be addressed in the study.

 Can the authors explain their methodological decisions? I appreciate the challenge in collecting online responses. But I think that the authors need to acknowledge that the number of participants is a serious limitation. The authors may need to explain why the data were analyzed in the way reported in the manuscript. How do the collection and analysis of data correspond to the research questions? I think that we need an explicit alignment between research questions and data collection/analysis.

 I also suggest that the authors presented the results as answers to the research questions. This means that they can use the research questions to organize the presentation of findings. I am not sure if the authors need to follow APA to format the statistical reporting. I also feel that the authors need to explain what these statistical results mean. The manuscript has a lot of tables but it is better for the authors to engage with them and interpret the results for readers (as responses to the research questions).

 While the discussion has important themes, I feel that the authors need to highlight the contributions that the study has made to the field. In what sense do they add to what we have already known about the topic? Then they can talk about what implications we can draw from the findings.

 I feel that the authors need to discuss the study’s methodological limitations.

Is the work clearly and accurately presented and does it cite the current literature?

Partly

If applicable, is the statistical analysis and its interpretation appropriate?

Partly

Are all the source data underlying the results available to ensure full reproducibility?

Yes

Is the study design appropriate and is the work technically sound?

Partly

Are the conclusions drawn adequately supported by the results?

Partly

Are sufficient details of methods and analysis provided to allow replication by others?

Partly

Reviewer Expertise:

education

I confirm that I have read this submission and believe that I have an appropriate level of expertise to confirm that it is of an acceptable scientific standard, however I have significant reservations, as outlined above.
